# Biomarkers for differentiation of coronavirus disease 2019 or extracorporeal membrane oxygenation related inflammation and bacterial/fungal infections in critically ill patients: A prospective observational study

**DOI:** 10.3389/fmed.2022.917606

**Published:** 2022-10-06

**Authors:** Matthias Weiss-Tessbach, Franz Ratzinger, Markus Obermueller, Heinz Burgmann, Thomas Staudinger, Oliver Robak, Monika Schmid, Bernhard Roessler, Bernd Jilma, Manuel Kussmann, Ludwig Traby

**Affiliations:** ^1^Division of Infectious Diseases and Tropical Medicine, Department of Medicine I, Medical University of Vienna, Vienna, Austria; ^2^Department of Clinical Pharmacology, Medical University of Vienna, Vienna, Austria; ^3^Ihr Labor, Medical Diagnostics Laboratories, Vienna, Austria; ^4^Department of Medicine I, Intensive Care Unit 13i2, Medical University of Vienna, Vienna, Austria; ^5^Division of Gastroenterology and Hepatology, Department of Medicine III, Medical University of Vienna, Vienna, Austria; ^6^Department of Anaesthesia, Intensive Care Medicine and Pain Medicine, Medical Simulation and Emergency Management Research Group, Medical University of Vienna, Vienna, Austria

**Keywords:** diagnosis differential, specificity and sensitivity, co-infection, interleukin-10, intensive care unit, extracorporeal membrane oxygenation (ECMO)

## Abstract

Secondary infections in coronavirus disease 2019 (COVID-19) patients are difficult to distinguish from inflammation associated with COVID-19 and/or extracorporeal membrane oxygenation (ECMO). Therefore, highly specific and sensitive biomarkers are needed to identify patients in whom antimicrobial therapy can be safely withheld. In this prospective monocentric study, 66 COVID-19 patients admitted to the intensive care unit (ICU) for ECMO evaluation were included. A total of 46 (70%) patients with secondary infections were identified by using broad microbiological and virological panels and standardized diagnostic criteria. Various laboratory parameters including C-reactive protein (CRP), interleukin (IL)-6, procalcitonin (PCT), and IL-10 were determined at time of study inclusion. The best test performance for differentiating bacterial/fungal secondary infections and COVID-19 and/or ECMO associated inflammation was achieved by IL-10 (ROC-AUC 0.84) and a multivariant step-wise regression model including CRP, IL-6, PCT, and IL-10 (ROC-AUC 0.93). Data obtained in the present study highlights the use of IL-10 to differentiate secondary bacterial/fungal infections from COVID-19 and/or ECMO associated inflammation in severely ill COVID-19 patients.

## Introduction

During the first wave in the United States, coronavirus disease 2019 (COVID-19) resulted in high rates of hospitalization (14%), intensive care unit (ICU) admissions (2%), and mortality (5%), leading to an extreme burden on the health care system (1). The very high mortality in European ICUs of about 30% did not change during the first three waves and therefore underscores the extreme burden of the COVID-19 pandemic on global health systems (2). In critically ill patients COVID-19 related pulmonary dysbiosis is a predisposing factor for development of secondary bacterial/fungal infections, occurring in 14–41% of ICU patients (3–5).

Influenced by recent guidelines of the Infectious Disease Society of America on the management of critically ill influenza patients, frequent antimicrobial usage of up to 86.4% has been observed at the beginning of the COVID-19 pandemic (6). This high use of antimicrobials was questioned, based on low rates of secondary infections observed as the pandemic progressed. Nevertheless, secondary bacterial/fungal infections led to increased mortality rates, highlighting the need for rapid and adequate identification of these patients (3).

To date, biomarkers such as white blood cell count (WBC), C-reactive protein (CRP), and procalcitonin (PCT) have been studied to distinguish secondary infections from COVID-19 related inflammation. However, one study could only exclude bacterial co-infections in 46%, while another study lacked sufficient microbiological diagnostics and/or clinical characterization of secondary bacterial/fungal infections (7, 8).

This limited potential of differentiation is further complicated in COVID-19 acute respiratory distress syndrome (ARDS) patients requiring extracorporeal membrane oxygenation (ECMO) support, by non-infectious activation of inflammatory pathways (9). A less studied parameter in this context is interleukin (IL)-10, whose production is stimulated by peptidoglycans of the bacterial cell wall and has been shown in previous studies to be a strong prognostic factor for mortality as well as the bacterial loads in blood of patients with *Staphylococcus aureus (S. aureus)* bacteremia (10–13).

The aim of the present study was to determine biomarkers that allow safe discontinuation of antimicrobial therapy by correctly identifying secondary bacterial/fungal infections in critically ill COVID-19 patients admitted to a specialized ECMO center for treatment of ARDS.

## Methods

### Study design

This prospective observational study included patients with a positive real-time polymerase chain reaction for Severe Acute Respiratory Syndrome Coronavirus type 2 (SARS-CoV-2) from nasopharyngeal swabs and/or bronchial lavage, who had ongoing antimicrobial therapy for at least 48 h and were admitted to an ICU at the University Hospital Vienna. Patients were included in the present study at the time of admission to the participating ICUs, which in most cases was to assess the need for ECMO support for underlying ARDS. Patients below the age of 18 years were excluded from study participation. At study inclusion, a diagnostic panel consisting of inflammatory parameters such as CRP, IL-6, PCT, WBC, microbiological, and radiological diagnostics was performed, and additional blood samples were directly frozen at −80°C until further processing (Supplementary Table 1).

### Interleukin-10 assay

Serum IL-10 (R&D Systems, Minneapolis, USA) was determined by sandwich enzyme immunoassays as recommended by the manufacturer, whereas all additional parameters were analyzed as part of routine diagnostics.

### Data collection

The Charlson comorbidity index, the Acute Physiology and Chronic Health Evaluation (APACHE) IV, Acute Physiology Score (APS), Sequential Organ Failure Assessment (SOFA), and Simplified Acute Physiology Score (SAPS-II) score were assessed at study inclusion. Disease severity was classified as mild, moderate, severe or critical according to the world health organization (WHO) severity classification.

The site of infection was classified according to the criteria of the European Centre for Disease Prevention and Control (ECDC) Point Prevalence Survey on Healthcare Associated Infections (version 5.3). The clinical, microbiological, and laboratory parameters necessary for this classification, collected within 48 h around admission, were obtained from the patient information system of the general hospital Vienna (14). Each patient was then discussed again in an expert panel consisting of three infectious disease specialists to interpret ambiguous microbiological results and thus select the most appropriate classification.

### Statistical analysis

Statistical analysis was performed using R Version 4.0.3 (Vienna, Austria). Categorical data are summarized as count with their percentage. Numeric data are presented as median with 1st and 3rd quartiles. The discriminatory ability of individual parameters was assessed using Wilcoxon rank tests and the area under the Receiver Characteristic Operator (ROC-AUC) curve. Cut-off points were calculated using the Youden Index method (pROC, R Package). Forward stepwise logistic regression models were established minimizing the Akaike information criterion (AIC) using age, sex, WHO-severity score, co-infection, and the laboratory parameters displayed in Supplementary Table 1. Additionally, a model with the two most discriminatory parameters was created. Due to the low number of available observations, cross-validation schemes or other methods for estimating the robustness of the predictive ability were not performed. Where appropriate, an accumulation of an alpha error related to multiple testing was controlled by the Bonferroni-Holm method.

Statistical significance was defined as *p*-values less than 0.05.

## Results

Patients’ demographics and disease related characteristics are shown in Table 1. A total of 66 patients with a median age of 54 years and ongoing antimicrobial therapy were included in this study. Overall 92% of patients were defined as critical according to WHO severity classification, with 55% receiving ECMO support, which was started on average 1 day (SD ± 1.2 days) before study inclusion.

**TABLE 1:** Patients characteristics and outcomes (*N* = 66).

Baseline characteristics	Bacterial/Fungal infections (*N* = 46)	No additional infection (*N* = 20)
Age—median years (IQR)	54 (48–62)	61 (54–66)
Female sex—*N* (%)	19 (41%)	7 (35%)
BMI—median (IQR)	32 (28–38)	29 (27–34)
Symptom onset to—median days (IQR)		
Hospital admission	6 (3–7)	3 (2–5)
ICU admission	7 (6–11)	7 (5–12)
Study inclusion	16 (12–20)	14 (11–21)
Supportive measures—*N* (%)		
Non-invasive ventilation^a^	0	3 (15%)
Invasive ventilation	46 (100%)	17 (85%)
Extracorporeal membrane oxygenation	26 (57%)	10 (50%)
**Scores at study inclusion**		
SOFA score at inclusion^b^—median (IQR)	8 (7–9)	7 (6–7)
SAPS2 score at inclusion^c^—median (IQR)	38 (30–47)	38 (32–50)
APACHE-IV score at inclusion^d^—median (IQR)	91 (77–99)	83 (71–95)
Estimated mortality rate at inclusion (APACHE-IV) in %	60 (49–70)	58 (40–68)
APS score at inclusion^d^—median (IQR)	81 (74–87)	75 (68–86)
COVID severity at inclusion^e^—N (%)		
Moderate	0	1 (5%)
Severe	2 (4%)	2 (10%)
Critical	44 (96%)	17 (85%)
Charlson comorbidity index^f^—*N* (%)		
0	9 (20%)	4 (20%)
1	19 (41%)	1 (5%)
2	7 (15%)	6 (30%)
3 +	11 (24%)	9 (45%)
**Co-morbidities—*N* (%)**		
Autoimmune disease^g^	4 (9%)	2 (10%)
Cardiovascular disease^h^	2 (4%)	4 (20%)
Chronic kidney disease	1 (2%)	1 (5%)
COPD	1 (2%)	3 (15%)
Diabetes mellitus	8 (17%)	5 (25%)
Hypertension	20 (43%)	11 (55%)
Malignancy^i^	4 (9%)	0
Post solid organ transplantation	0	1 (5%)
Others^j^	8 (17%)	5 (25%)

IQR, Interquartile range; BMI, Body Mass Index; ICU, Intensive care unit; SOFA, Sequential Organ Failure Assessment; SAPS, Simplified Acute Physiology Score; APACHE, Acute Physiology And Chronic Health Evaluation; APS, Admission Point Score; COVID, Coronavirus disease; COPD, Chronic obstructive pulmonary disease.

^*a*^Non-invasive ventilation included patients receiving oxygen *via* high flow nasal cannula.

^*b*^SOFA score was calculated according to Vincent et al. (29).

^*c*^SAPS2 was calculated according to Le Gall et al. (30).

^*d*^APS and APACHE-IV was calculated according to Zimmerman et al. (31).

^*e*^Disease severity was classified according to the WHO (32) severity classification. WHO reference number: WHO/2019-nCoV/clinical/2021.1.

^*f*^Charlson comorbidity index was calculated according to Quan et al. (33).

^*g*^Autoimmune diseases included psoriasis, rheumatoid arthritis, lupus erythematodes, vasculitis and myasthenia gravis.

^*h*^Cardiovascular disease included cardiac insufficiency, coronary artery disease, and myocardial infarction, cerebrovascular accident.

^*i*^Malignancy was defined as an active and ongoing solid or hematogenous neoplasia.

^*j*^Others included liver disease, defined as cirrhosis with or without portal hypertension, peptic ulcer disease and hypothyroidism.

Secondary infections were observed in 46 patients at time of study inclusion. Out of these, 26 patients had monomicrobial (21 bacterial, 5 fungal) and 11 patients polymicrobial infections. Nine patients were classified as systemic infections (SYS) according to the ECDC criteria as no causative pathogen could be detected, clinical sings of an acute infection were present and the physician instituted treatment for sepsis. The most common infection sources were pulmonary (*n* = 26, 57%), catheter related (*n* = 5, 11%) and urinary tract infections (*n* = 2, 4%). Additionally, blood stream infections of unknown origin and SYS without detected causative pathogens were found in 2 (4%) and 9 (20%) patients, respectively. The overall mortality was 38% (*n* = 25) with a 28-day mortality of 15% (*n* = 10) and a median hospital stay of 47 days (Supplementary Table 1).

When comparing patients with and without ECMO support, no apparent differences in levels of CRP, IL-6, IL-10, PCT, lactate dehydrogenase (LDH), D-dimer, leukocytes, fibrinogen, ferritin, the neutrophils/lymphocyte ratio, the IL-6/IL-10 ratio, and the SOFA score were observed (Supplementary Figure 1).

**FIGURE 1:**
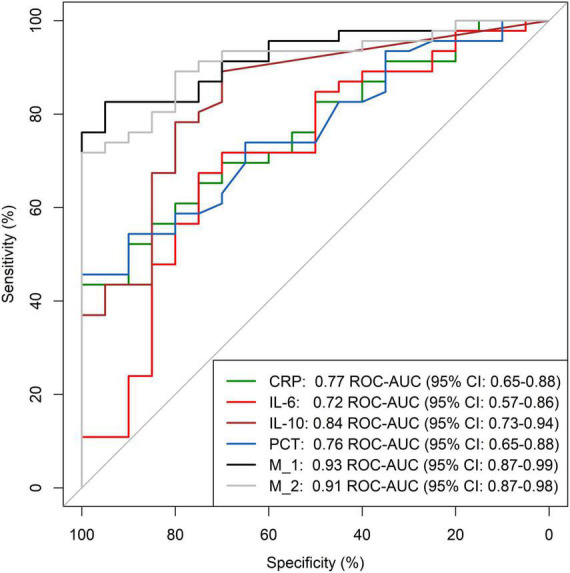
ROC analysis for detection of additional infections in COVID-19 ICU patients. ROC-AUC, area under the receiver operating characteristics curve; 95% CI, 95% Confidence interval; CRP, C-reactive Protein; IL-6, Interleukin-6; IL-10, Interleukin-10; PCT, Procalcitonin; M_1 (model 1) and M_2 (model 2), multivariate step-wise regression models. M_1 included CRP, IL-6, IL-10 and procalcitonin. M_2 included CRP and IL-10.

The most discriminatory parameters for secondary bacterial/fungal infections (*p*-value, ROC-AUC, 95% CI) were CRP (*p* = 0.0005, 0.77, 0.65–0.88), IL-10 (*p* < 0.0001, 0.88, 0.73–0.94), and PCT (*p* = 0.0008, 0.76, 0.65–0.88) (Supplementary Table 2). A forward step-wise logistic regression model was calculated resulting in a ROC-AUC of 0.93 (95% CI: 0.87–0.99). The final model included CRP, IL-6, IL-10, and PCT (model 1). A model solely based on CRP and IL-10 (model 2) resulted in a ROC-AUC of 0.91 (95% CI: 0.87–0.98) (Figure 1). However, both logistic regression models were not superior at predicting the presence of superinfections compared to IL-10 alone (*p* = 0.154, *p* = 0.263). IL-10 showed the best sensitivity (96%) and specificity (100%) at a cut-off value of 15.4 pg/ml compared to CRP, IL-6 and PCT (Supplementary Table 2). When a correlation matrix of major biomarkers and clinical scores was calculated, apart from the APS with the APACHE-IV score, there was no strong correlation, even between commonly used inflammation parameters (Supplementary Figure 2).

## Discussion

In critically ill COVID-19 patients predominantly admitted for ECMO evaluation, IL-10 supported the discrimination of bacterial/fungal secondary infections from inflammation caused by COVID-19 and/or ECMO. IL-10 alone could rule out secondary bacterial/fungal infections with an negative predictive value (NPV) of 89% (cut-off: 15.4 ng/mL), providing intensivists with a much needed tool to avoid unnecessary antibiotic therapy and to treat actual secondary infections as soon as possible.

The present study investigated a well-defined patient population with high rates of standardized and tailored microbiological/virological diagnostics obtained upon study inclusion (Supplementary Table 3). Comparable studies either focused on specific infections like pneumonia, showed lower rates of microbiological diagnostics (17–64%) due to a retrospective study design or have not stated their microbiological workup (15–17). Based on frequent microbiological diagnostics and the specific study population, including high rates of patients on ECMO (55%), high rates of secondary infections (70%), characterized using well defined ECDC criteria, were observed in the present study.

Previous reports in different ICU cohorts demonstrating lower morbidity severity indices (e.g., APACHE IV and SOFA score) with no or low rates (9.5%) of patients on ECMO observed secondary infections in 21–69% (16, 18–20). Although the study by De Bruyn et al. included less severely ill COVID-19 patients and markedly reduced microbiological diagnostics, it also showed high rates of secondary infections (69%), with pneumonia (57%), bloodstream infections of unknown origin (30%), and catheter-related sepsis (15%) being the most common sites of infection.

Acute COVID-19 infection is associated with inflammatory states and may be followed by a prolonged immune system dysregulation including consistently elevated inflammatory markers like IL-6, ferritin or CRP, for several weeks (21). ECMO therapy, a corner stone in the management of severe COVID-19 ARDS, increases inflammatory markers like WBC, IL-6, IL-10, and tumor necrosis factor (TNF)-alpha due to activation of inflammatory and coagulation pathways caused by the constant contact of cellular and humoral blood components on the large extracorporeal surface (9).

COVID-19 and/or ECMO induced inflammation might therefore complicate diagnosis of secondary bacterial/fungal infections, frequently supported by well-established laboratory markers. Various biomarkers were investigated to distinguish COVID-19 from bacterial infections such as CRP and/or WBC. A combination of WBC at admission and trajectories of CRP over 72 h allowed to exclude 46% of bacterial co-infections in COVID-19 patients (8). Another study investigated the outcome of COVID-19 patients newly admitted to the hospital with a PCT < 0.25 ng/mL, resulting in lower rates of antimicrobial prescription, ICU admission and 28-day mortality (7).

Previous studies investigated the usage of CRP and PCT to guide antimicrobial therapy (15). In a collective of 66 ICU patients secondary infections occurred in 50%, PCT demonstrated a positive predictive value (PPV) of 93% and a NPV of 81% with a cutoff of > 1 and < 0.25 μg/L, respectively. The present study showed a NPV of 77% and a PPV of 100% using a PCT cut-off of 0.6 ng/mL (Supplementary Table 2). Although van Berkel et al. investigated a less severely ill patient population, indicated by the lack of ECMO support, CRP and PCT showed comparable ROC-AUC values of 0.76 and 0.8, respectively, when compared to the present study (15).

In addition, Pink et al. showed higher ROC-AUC values for CRP (0.86) and PCT (0.88) for discrimination of secondary infections in a mixed patients collective consisting of 52 ICU and 47 non-ICU COVID-19 patients (22). However, the inclusion of non-ICU patients, the lack of ECMO support and the low rates of vasopressor use limit comparability with the present study.

IL-10 is mainly known as an anti-inflammatory cytokine and was shown to be a prognostic marker for poor outcome in COVID-19 patients. While the exact mechanisms are still unclear, evidence points in either a pro-inflammatory effect of high IL-10 levels causing T-cell exhaustion and/or an IL-10 “resistance” which is entangled with hyperglycemia (23). IL-10 concentrations observed in the present study (median: 13 pg/mL) were comparable to published data in patients with *S. aureus* bacteremia (median: 10–20 pg/mL) (24).

This association of IL-10 with poor outcomes in COVID-19 patients might be at least partly explained by secondary infections. IL-10 was the single parameter with the highest discriminatory power compared with CRP, IL-6 and PCT, which was exceeded only by using combinations of four markers (Model 1: CRP, IL-6, IL10, PCT; ROC-AUC 0.93) or CRP and IL-10 (Model 2: ROC-AUC 0.91) in two logistic regression models. In previous studies, elevated IL-10 at time of hospital admission or up to 72 h after presentation was shown to be predictive for mortality in patients with *S. aureus* bacteremia (13, 25). Although the exact reason for the association between increased IL-10 and increased mortality is not known, it has been demonstrated that IL-10 production is stimulated by components of the bacterial cell wall, particularly peptidoglycans, and is dependent on antigen levels and thus bacterial load (11, 12). Therefore, the increased mortality rate could be due to delayed or inadequate treatment of bacterial infections, so that the bacterial load in the blood, and consequently IL-10, remained significantly elevated. In conjunction with the data obtained in this study, IL-10 may thus be a valuable marker for a variety of infections, not only *S. aureus* bacteremia, and may potentially aid in clinical diagnosis finding. In addition, previous data investigating IL-10 serum concentrations in patients with *S. aureus* bacteremia demonstrated no significant change within the first 72 h, even with targeted antimicrobial treatment (24).

Interestingly, in this study, no differences in inflammatory parameters were detected between patients with and without ECMO support, although previous literature has described activation of inflammatory pathways by the extracorporeal surface during ECMO therapy. This lack of differences could be explained by the inflammation already triggered by COVID-19. However, further studies examining patients on ECMO support with and without COVID-19 are needed for a definitive explanation.

One of the main limitations of this study is the variability regarding the time of symptom onset to ICU admission, study inclusion and ECMO therapy start. This may lead to variations in the initial inflammatory stimulus, which, in conjunction with different production times and half-lives of the biomarkers studied, may explain the lack of correlation of frequently used inflammatory parameters with each other and with IL-10 (11, 26–28). Nevertheless, this study represents a real life scenario of a specialized center experienced in ARDS treatment and ECMO support, providing care of patients directly admitted from the emergency department, but also from external ICUs due to worsening respiratory failure.

This data emphasizes the use of IL-10 alone or in combination with CRP, IL-6 and PCT, to distinguish secondary infections from COVID-19 associated inflammation severely and critically ill COVID-19 patients. This biomarker may allow clinicians to omit unnecessary antimicrobial therapy. This hypothesis generating study warrants further interventional trials to evaluate the feasibility and safety of biomarker based use of antiinfectives.

## Data availability statement

The original contributions presented in the study are included in this article/Supplementary material, further inquiries can be directed to the corresponding author/s.

## Ethics statement

The studies involving human participants were reviewed and approved by the Ethics Commitee of the Medical University of Vienna, Vienna, Austria (No. 1404/2020). The patients or legal representatives provided their written informed consent to participate in this study if possible. If this was not feasible at the time of enrollment, informed consent was obtained from patients or their legal representatives at a later time if possible.

## Author contributions

MWT, LT, and MKu wrote the manuscript and participated in the infectious disease panel. MWT gathered the patient data and performed the experiments. LT and MKu designed the study. HB, BJ, MO, FR, and OR critically read and revised the manuscript. MO performed additional laboratory analysis. FR performed the statistical analysis. TS, OR, MS, BR, and LT treated and recruited the patients. All authors read and approved the final manuscript.

## Supplementary material

SUPPLEMENTARY FIGURE 1 Comparison of different biomarkers between patient with and without ECMO support. Figures consist of individual data points, the median and 95% confidence interval. Solitary data points outside of figure scales were set as maximum scale values and are shown as (X). For IL-10, procalcitonin and IL6/IL10 ratio, samples below the limit of detection were set as 0.1 for graphical representation. CRP, C-reactive protein; IL-6, Interleukin-6; IL-10, Interluekin-10; LDH, Lactate Dehydrogenase.

SUPPLEMENTARY FIGURE 2 Heat map of correlation matrix between different biomarkers and clinical scores. The correlation is indicated *via* a color scale proportional to its strength of association, ranging from blue (negative correlation; negative values) to red (positive correlation; positive values). Each cell contains the pair-wise spearman correlation coefficient between the respective biomarkers from the overall population analyzed. PTZ, prothrombin time; INR, international normalized ratio; APTT, activated partial thromboplastin time; ASAT, aspartate transaminase; ALAT, alanine transaminase; LDH, lactate dehydrogenase; CRP, C-reactive protein; IL-6, interleukin 6; IL-10, interleukin 10.

SUPPLEMENTARY TABLE 1 Table of raw patient data.

SUPPLEMENTARY TABLE 2 Laboratory parameters for detection of secondary infections in COVID-19 ICU patients.

SUPPLEMENTARY TABLE 3 Table of microbiological tests performed within 48 h of study inclusion.

## References

[1] Stokes EK. Coronavirus Disease 2019 case surveillance — United States, January 22–May 30, 2020. *MMWR Morb Mortal Wkly Rep.* (2020) 69:759–65. 10.15585/mmwr.mm6924e2 32555134 PMC7302472

[2] Carbonell R Urgelés S Rodríguez A Bodí M Martín-Loeches I Solé-Violán J Mortality comparison between the first and second/third waves among 3,795 critical COVID-19 patients with pneumonia admitted to the ICU: a multicentre retrospective cohort study. *Lancet Reg Health Eur.* (2021) 11:100243. 10.1016/j.lanepe.2021.100243 34751263 PMC8566166

[3] Lansbury L Lim B Baskaran V Lim WS. Co-infections in people with COVID-19: a systematic review and meta-analysis. *J Infect.* (2020) 81:266–75. 10.1016/j.jinf.2020.05.046 32473235 PMC7255350

[4] Musuuza JS Watson L Parmasad V Putman-Buehler N Christensen L Safdar N. Prevalence and outcomes of co-infection and superinfection with SARS-CoV-2 and other pathogens: a systematic review and meta-analysis. *PLoS One.* (2021) 16:e0251170. 10.1371/journal.pone.0251170 33956882 PMC8101968

[5] Hernández-Terán A Mejía-Nepomuceno F Herrera MT Barreto O García E Castillejos M Dysbiosis and structural disruption of the respiratory microbiota in COVID-19 patients with severe and fatal outcomes. *Sci Rep.* (2021) 11:21297. 10.1038/s41598-021-00851-0 34716394 PMC8556282

[6] Uyeki TM Bernstein HH Bradley JS Englund JA File TM Fry AM Clinical practice guidelines by the infectious diseases society of America: 2018 update on diagnosis, treatment, chemoprophylaxis, and institutional outbreak management of seasonal influenza. *Clin Infect Dis.* (2019) 68:e1–47. 10.1093/cid/ciy866 30566567 PMC6653685

[7] Williams EJ Mair L de Silva TI Green DJ House P Cawthron K Evaluation of procalcitonin as a contribution to antimicrobial stewardship in SARS-CoV-2 infection: a retrospective cohort study. *J Hosp Infect.* (2021) 110:103–7. 10.1016/j.jhin.2021.01.006 33484783 PMC7817391

[8] Mason CY Kanitkar T Richardson CJ Lanzman M Stone Z Mahungu T Exclusion of bacterial co-infection in COVID-19 using baseline inflammatory markers and their response to antibiotics. *J Antimicrob Chemother.* (2021) 76:1323–31. 10.1093/jac/dkaa563 33463683 PMC7928909

[9] Kowalewski M Fina D Słomka A Raffa GM Martucci G Lo Coco V COVID-19 and ECMO: the interplay between coagulation and inflammation—a narrative review. *Crit Care.* (2020) 24:205. 10.1186/s13054-020-02925-3 32384917 PMC7209766

[10] Matsui K Ikeda R. Peptidoglycan in combination with muramyldipeptide synergistically induces an interleukin-10-dependent T helper 2-dominant immune response. *Microbiol Immunol.* (2014) 58:260–5. 10.1111/1348-0421.12139 24479522

[11] Frodermann V Chau TA Sayedyahossein S Toth JM Heinrichs DE Madrenas JA. Modulatory interleukin-10 response to staphylococcal peptidoglycan prevents Th1/Th17 adaptive immunity to *Staphylococcus aureus*. *J Infect Dis.* (2011) 204:253–62. 10.1093/infdis/jir276 21673036

[12] Rose WE Shukla SK Berti AD Hayney MS Henriquez KM Ranzoni A Increased endovascular *Staphylococcus aureus* inoculum is the link between elevated serum interleukin 10 concentrations and mortality in patients with bacteremia. *Clin Infect Dis.* (2017) 64:1406–12. 10.1093/cid/cix157 28205673 PMC5411397

[13] Rose WE Eickhoff JC Shukla SK Pantrangi M Rooijakkers S Cosgrove SE Elevated serum interleukin-10 at time of hospital admission is predictive of mortality in patients with *Staphylococcus aureus* bacteremia. *J Infect Dis.* (2012) 206:1604–11. 10.1093/infdis/jis552 22966128 PMC6281403

[14] European Centre for Disease Prevention and Control. *Point prevalence survey of healthcare-associated infections and antimicrobial use in European acute care hospitals: protocol version 5.3: ECDC PPS 2016–2017.* (2016). Available online at: https://data.europa.eu/doi/10.2900/374985 (Accessed June 18, 2021).

[15] van Berkel M Kox M Frenzel T Pickkers P Schouten J RCI-COVID-19 Study Group Biomarkers for antimicrobial stewardship: a reappraisal in COVID-19 times? *Crit Care.* (2020) 24:600. 10.1186/s13054-020-03291-w 33023606 PMC7538269

[16] Pickens CO Gao CA Cuttica MJ Smith SB Pesce LL Grant RA Bacterial superinfection pneumonia in patients mechanically ventilated for COVID-19 pneumonia. *Am J Respir Crit Care Med.* (2021) 204:921–32. 10.1164/rccm.202106-1354OC 34409924 PMC8534629

[17] Russell CD Fairfield CJ Drake TM Turtle L Seaton RA Wootton DG Co-infections, secondary infections, and antimicrobial use in patients hospitalised with COVID-19 during the first pandemic wave from the ISARIC WHO CCP-UK study: a multicentre, prospective cohort study. *Lancet Microbe.* (2021) 2:e354-65. 10.1016/S2666-5247(21)00090-2 PMC8172149 34100002

[18] Bardi T Pintado V Gomez-Rojo M Escudero-Sanchez R Azzam Lopez A Diez-Remesal Y Nosocomial infections associated to COVID-19 in the intensive care unit: clinical characteristics and outcome. *Eur J Clin Microbiol Infect Dis.* (2021) 40:495–502. 10.1007/s10096-020-04142-w 33389263 PMC7778834

[19] De Bruyn A Verellen S Bruckers L Geebelen L Callebaut I De Pauw I Secondary infection in COVID-19 critically ill patients: a retrospective single-center evaluation. *BMC Infect Dis.* (2022) 22:207. 10.1186/s12879-022-07192-x 35236299 PMC8890021

[20] Zhang H Zhang Y Wu J Li Y Zhou X Li X Risks and features of secondary infections in severe and critical ill COVID-19 patients. *Emerg Microbes Infect.* (2020) 9:1958–64. 10.1080/22221751.2020.1812437 32815458 PMC8284966

[21] Patel P DeCuir J Abrams J Campbell AP Godfred-Cato S Belay ED. Clinical characteristics of multisystem inflammatory syndrome in adults: a systematic review. *JAMA Netw Open.* (2021) 4:e2126456. 10.1001/jamanetworkopen.2021.26456 34550381 PMC8459192

[22] Pink I Raupach D Fuge J Vonberg R-P Hoeper MM Welte T C-reactive protein and procalcitonin for antimicrobial stewardship in COVID-19. *Infection.* (2021) 49:935–43. 10.1007/s15010-021-01615-8 34021897 PMC8140571

[23] Islam H Chamberlain TC Mui AL Little JP. Elevated interleukin-10 levels in COVID-19: potentiation of pro-inflammatory responses or impaired anti-inflammatory action? *Front Immunol.* (2021) 12:677008. 10.3389/fimmu.2021.677008 34234779 PMC8255680

[24] Volk CF Burgdorf S Edwardson G Nizet V Sakoulas G Rose WE. Interleukin (IL)-1β and IL-10 host responses in patients with *Staphylococcus aureus* bacteremia determined by antimicrobial therapy. *Clin Infect Dis.* (2020) 70:2634–40. 10.1093/cid/ciz686 31365924 PMC7286365

[25] Minejima E Bensman J She RC Mack WJ Tran MT Ny P A dysregulated balance of pro- and anti-inflammatory host cytokine response early during therapy predicts persistence and mortality in *Staphylococcus aureus* bacteremia. *Crit Care Med.* (2016) 44:671–9. 10.1097/CCM.0000000000001465 26540400 PMC6504958

[26] Weidhase L Wellhöfer D Schulze G Kaiser T Drogies T Wurst U Is interleukin-6 a better predictor of successful antibiotic therapy than procalcitonin and C-reactive protein? A single center study in critically ill adults. *BMC Infect Dis.* (2019) 19:150. 10.1186/s12879-019-3800-2 30760225 PMC6375140

[27] Schuetz P Albrich W Mueller B. Procalcitonin for diagnosis of infection and guide to antibiotic decisions: past, present and future. *BMC Med.* (2011) 9:107. 10.1186/1741-7015-9-107 21936959 PMC3186747

[28] Schmit X Vincent JL. The time course of blood c-reactive protein concentrations in relation to the response to initial antimicrobial therapy in patients with sepsis. *Infection.* (2008) 36:213–9. 10.1007/s15010-007-7077-9 18463788

[29] Vincent JL Moreno R Takala J Willatts S De Mendonça A Bruining H The SOFA (Sepsis-related Organ Failure Assessment) score to describe organ dysfunction/failure. On behalf of the working group on sepsis-related problems of the european society of intensive care medicine. *Intensive Care Med.* (1996) 22:707-710. 10.1007/BF01709751 8844239

[30] Le Gall JR Lemeshow S Saulnier F. A new simplified acute physiology score (SAPS II) based on a European/North American multicenter study. *JAMA.* (1993) 270:2957-2963. 10.1001/jama.270.24.2957 8254858

[31] Zimmerman JE Kramer AA McNair DS Malila FM. Acute physiology and chronic health evaluation (APACHE) IV: hospital mortality assessment for today’s critically ill patients. *Crit Care Med.* (2006) 34:1297-1310. 10.1097/01.CCM.0000215112.84523.F0 16540951

[32] World Health Organization [WHO]. *COVID-19 clinical management: living guidance, 25 January 2021. Document No: WHO/2019-nCoV/clinical/2021.1.* Geneva: World Health Organization (2021).

[33] Quan H Li B Couris CM Fushimi K Graham P Hider P Updating and validating the Charlson comorbidity index and score for risk adjustment in hospital discharge abstracts using data from 6 countries. *Am J Epidemiol.* (2011) 173:676-682. 10.1093/aje/kwq433 21330339

