# Supplementary figures and images for: Biomarkers for differentiation of coronavirus disease 2019 or extracorporeal membrane oxygenation related inflammation and bacterial/fungal infections in critically ill patients: A prospective observational study
Source: Front Med (Lausanne). 2022 Oct 6;9:917606. doi: 10.3389/fmed.2022.917606 (PMC9582266; doi:10.3389/fmed.2022.917606)

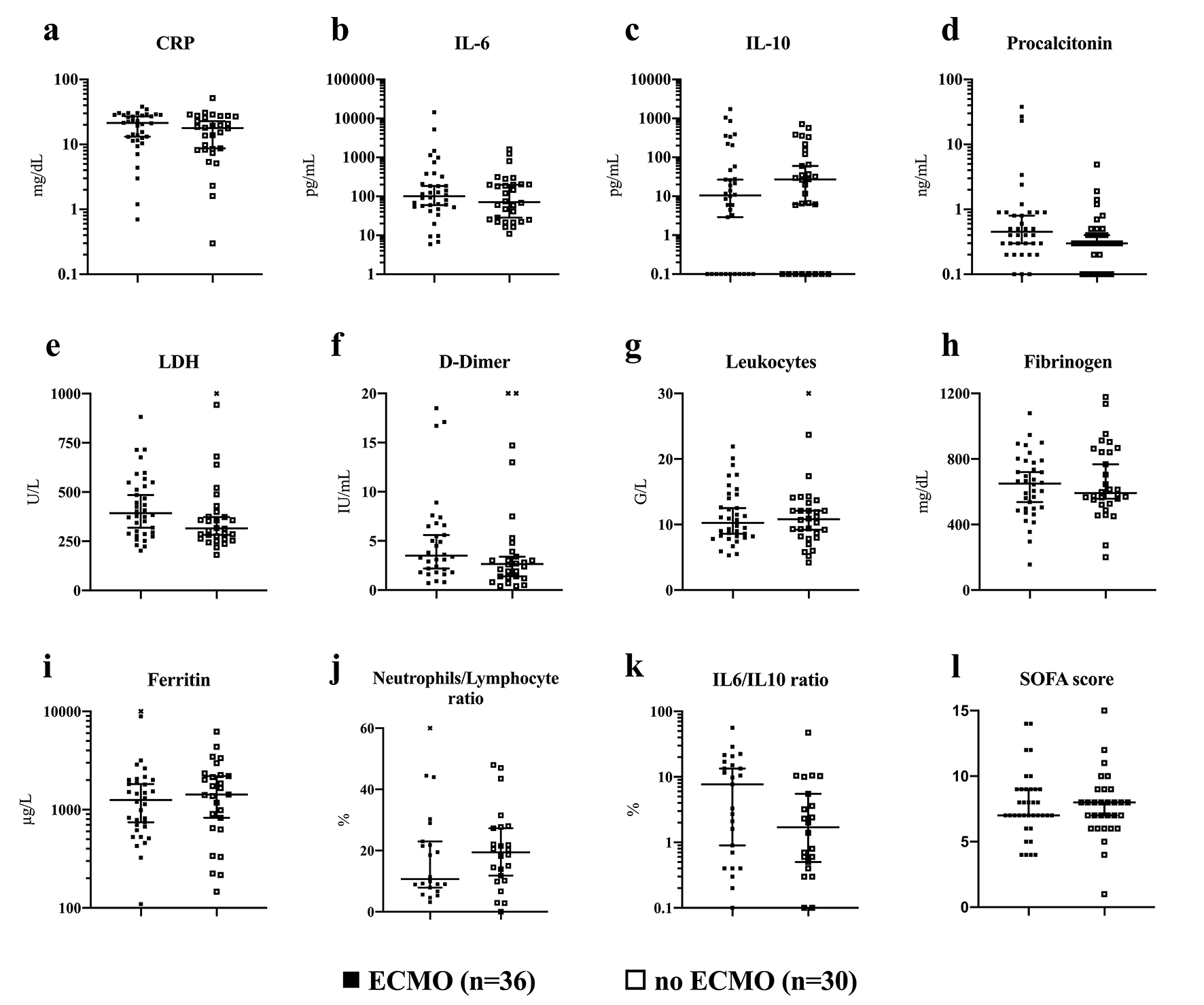

Supplement: SUPPLEMENTARY FIGURE 1 — Comparison of different biomarkers between patient with and without ECMO support. Figures consist of individual data points, the median and 95% confidence interval. Solitary data points outside of figure scales were set as maximum scale values and are shown as (X). For IL-10, procalcitonin and IL6/IL10 ratio, samples below the limit of detection were set as 0.1 for graphical representation. CRP, C-reactive protein; IL-6, Interleukin-6; IL-10, Interluekin-10; LDH, Lactate Dehydrogenase. [file Image_1.TIFF]

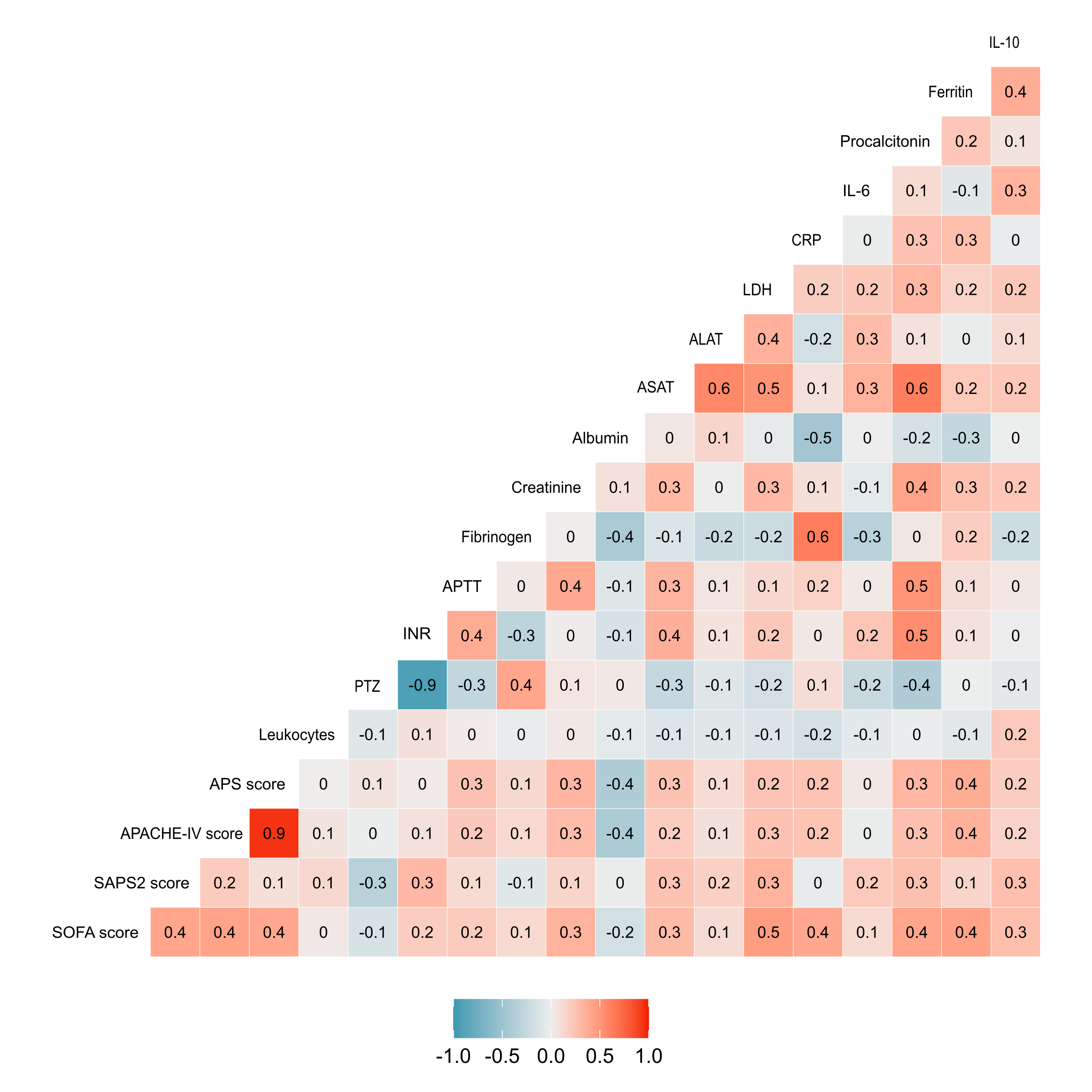

Supplement: SUPPLEMENTARY FIGURE 2 — Heat map of correlation matrix between different biomarkers and clinical scores. The correlation is indicated via a color scale proportional to its strength of association, ranging from blue (negative correlation; negative values) to red (positive correlation; positive values). Each cell contains the pair-wise spearman correlation coefficient between the respective biomarkers from the overall population analyzed. PTZ, prothrombin time; INR, international normalized ratio; APTT, activated partial thromboplastin time; ASAT, aspartate transaminase; ALAT, alanine transaminase; LDH, lactate dehydrogenase; CRP, C-reactive protein; IL-6, interleukin 6; IL-10, interleukin 10. [file Image_2.PNG]
