# Supplementary material for: Biomarkers for differentiation of coronavirus disease 2019 or extracorporeal membrane oxygenation related inflammation and bacterial/fungal infections in critically ill patients: A prospective observational study
Source: Front Med (Lausanne). 2022 Oct 6;9:917606. doi: 10.3389/fmed.2022.917606 (PMC9582266; doi:10.3389/fmed.2022.917606)
Supplement: SUPPLEMENTARY TABLE 2 — Laboratory parameters for detection of secondary infections in COVID-19 ICU patients. [file Table_2.DOCX]

**Supplementary Table 2: Laboratory parameters for detection of secondary infections in COVID-19 ICU patients**

|  | laboratory parameters | | | | | | | | | | | | logistic regression models | | | | | |  |
| --- | --- | --- | --- | --- | --- | --- | --- | --- | --- | --- | --- | --- | --- | --- | --- | --- | --- | --- | --- |
|  | **CRP (mg/l)** | | | **IL-6 (pg/ml)** | | | **procalcitonin (ng/ml)** | | | **IL-10 (pg/ml)** | | | **model 1 (CRP, IL-6, IL-10, PCT)** | | | **model 2 (CRP, IL-10)** | | |  |
| all patients (n=66) – median (IQR) | 20 (10-27) | | | 92 (41-203) | | | 0.4 (0.26-0.79) | | | 13 (0-64) | | |  | | |  | | |  |
| bacterial/fungal infections (n=46) – median (IQR) | 23 (13-29) | | | 134 (55-263) | | | 0.5 (0.28-0.91) | | | 27 (7-222) | | |  | | |  | | |  |
| no additional infection (n=20) – median (IQR) | 12 (7-18) | | | 50 (21-80) | | | 0.28 (0.13-0.39) | | | 0 (0-6) | | |  | | |  | | |  |
| ROC-AUC (95% CI) | 0.77 (0.65-0.88) | | | 0.72 (0.57-0.86) | | | 0.76 (0.65-0.88) | | | 0.84 (0.73-0.94) | | | 0.93 (0.87-0.99) | | | 0.91 (0.87-0.98) | | |  |
| sensitivity, specificity and predictive values | | | | | | | | | | | | | | | | | | |  |
| concentration cut-off | 10.6 | 21 | 27.1 | 25.3 | 68.1 | 163 | 0.2 | 0.44 | 0.6 | 1.6 | 6 | 15.4 |  |  |  |  |  |  | |
| sensitivity | 35% | 59% | 89% | 50% | 72% | 93% | 37% | 57% | 93% | 61% | 85% | 96% | 70% | 83% | 96% | 63% | 83% | 98% | |
| specificity | 55% | 95% | 100% | 45% | 75% | 95% | 60% | 100% | 100% | 60% | 80% | 100% | 85% | 100% | 100% | 75% | 100% | 100% | |
| negative predictive value | 40% | 49% | 73% | 41% | 54% | 80% | 41% | 49% | 77% | 49% | 70% | 89% | 59% | 70% | 89% | 54% | 69% | 94% | |
| positive predictive value | 82% | 98% | 100% | 78% | 87% | 97% | 83% | 100% | 100% | 83% | 90% | 100% | 94% | 100% | 100% | 90% | 100% | 100% | |

Definition of abbreviations: IQR: Interquartile range, ROC-AUC: area under the receiver operating characteristics curve, CRP: C-reactive protein, IL: Interleukin, Model 1 included CRP, IL-6, IL-10 and procalcitonin. Model 2 included CRP and IL-10. Optimal cut-off points were calculated using the Youden Index method (pROC, R Package).
